# Human Cytomegalovirus Inhibits the PARsylation Activity of Tankyrase—A Potential Strategy for Suppression of the Wnt Pathway

**DOI:** 10.3390/v8010008

**Published:** 2015-12-29

**Authors:** Sujayita Roy, Fengjie Liu, Ravit Arav-Boger

**Affiliations:** Department of Pediatrics, Johns Hopkins University School of Medicine, Baltimore 21287, MD, USA; sroy16@jhu.edu (S.R.); liufengjie2003@gmail.com (F.L.)

**Keywords:** Human cytomegalovirus (HCMV), tankyrase, Axin1, PARsylation, ubiquitination, Wnt/β-catenin

## Abstract

Human cytomegalovirus (HCMV) was reported to downregulate the Wnt/β-catenin pathway. Induction of Axin1, the negative regulator of the Wnt pathway, has been reported as an important mechanism for inhibition of β-catenin. Since Tankyrase (TNKS) negatively regulates Axin1, we investigated the effect of HCMV on TNKS expression and poly-ADP ribose polymerase (PARsylation) activity, during virus replication. Starting at 24 h post infection, HCMV stabilized the expression of TNKS and reduced its PARsylation activity, resulting in accumulation of Axin1 and reduction in its PARsylation as well. General PARsylation was not changed in HCMV-infected cells, suggesting specific inhibition of TNKS PARsylation. Similarly, treatment with XAV939, a chemical inhibitor of TNKS’ activity, resulted in the accumulation of TNKS in both non-infected and HCMV-infected cell lines. Reduction of TNKS activity or knockdown of TNKS was beneficial for HCMV, evidenced by its improved growth in fibroblasts. Our results suggest that HCMV modulates the activity of TNKS to induce Axin1, resulting in inhibition of the β-catenin pathway. Since HCMV replication is facilitated by TNKS knockdown or inhibition of its activity, TNKS may serve as an important virus target for control of a variety of cellular processes.

## 1. Introduction

Infection with human cytomegalovirus (HCMV), a β-herpesvirus, is near universal in humans and can cause significant morbidity and mortality in immunocompromised hosts, such as organ and bone marrow transplant recipients, and patients with AIDS [1]. HCMV is also the most common infectious cause of congenital birth defects [1]. Upon infection, HCMV manipulates and controls the host cell to facilitate its successful replication by dysregulating cell-signaling events [2]. An important signaling pathway hijacked by herpesviruses is the canonical Wnt/β-catenin pathway [3]. This pathway is critical for normal embryonic development and cellular differentiation and is the subject of investigation of numerous developmental and disease models [4]. Altered modulation of the Wnt pathway is a characteristic of multiple cancers and virus infections [3,5]. The key feature of the Wnt pathway is degradation of the downstream modulator β-catenin by the so-called “destruction complex” in the cytoplasm. The destruction complex is composed of a group of proteins: adenomatous polyposis coli (APC), glycogen-synthase kinase 3 (GSK3α, β) and Axin [6,7,8,9]. Wnt activation dissociates this complex and releases β-catenin which translocates into the nucleus and regulates the transcription of Wnt-responsive genes [10]. Axin1 is the focal point of control of the destruction complex; the concentration of Axin1 is the rate-limiting step in formation of the destruction complex [11]. Thus, modulation of Axin1 is a critical step in the outcome of Wnt signaling. Axin1 stabilization and degradation is subject to post-translational modifications, ubiquitination and PARsylation [12]. PARsylation of proteins is accomplished by poly-ADP ribose polymerases (PARPs), a group of enzymes that modify their target proteins (including themselves) post-translationally by catalyzing covalent addition of adenosine diphosphate (ADP) ribose units from nicotinamide adenine dinucleotide (NAD) to give rise to branched ADP-ribose chains on the acceptor protein. Axin1 is specifically PARsylated by Tankyrase 1 and 2 (also called PARP 5a and 5b) which promotes Axin1 degradation through the ubiquitin proteasome pathway [12].

Unlike the γ-herpesviruses (Epstein Barr virus, EBV and Kaposi sarcoma Herpesvirus, KSHV) and α-herpesviruses HSV1 and HSV2, we and others have reported that HCMV inhibits the Wnt/β-catenin pathway [13,14]. HCMV infection of human foreskin fibroblasts induced Axin1, the negative regulator of Wnt, resulting in decreased expression of β-catenin. To better understand strategies used by HCMV to inhibit Wnt/β-catenin signaling, we investigated whether HCMV modulated TNKS. Our results show that the upregulation of Axin1 follows an increase in TNKS stabilization in HCMV-infected cells. This apparent increase in TNKS protein level is a result of reduced PARsylation activity of TNKS, because TNKS also autoregulates its own stability through PARsylation. The inhibitory effect on the enzymatic activity of TNKS may be beneficial for HCMV, because a chemical inhibitor of TNKS (XAV939), or the knockdown of TNKS facilitated its replication. Therefore, inhibition of TNKS activity is a viral strategy for efficient replication and may contribute to inhibition of the Wnt pathway by HCMV.

## 2. Results

### 2.1. Human Cytomegalovirus (HCMV) Infection Stabilizes TNKS

**Figure 1:**
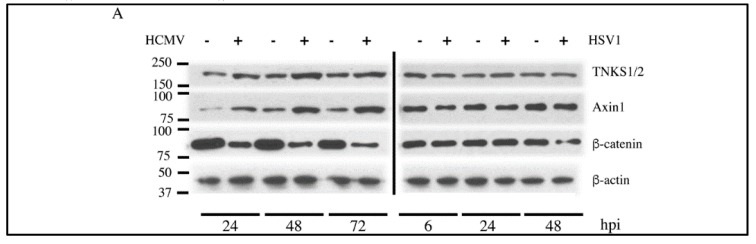

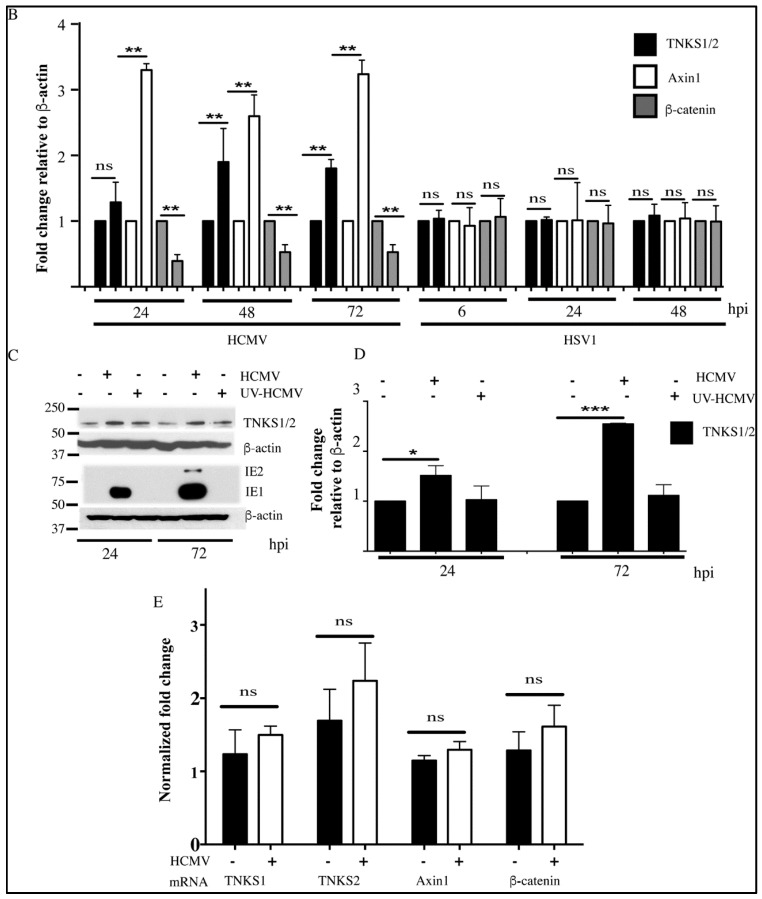
TNKS is induced by human cytomegalovirus (HCMV) but not herpesvirus (HSV) infection. (**A**) Western blot showing protein profile in response to HCMV and HSV infection at the indicated hpi. HFFs were infected with HCMV Towne or HSV1 at multiplicity of infection (MOI = 1) and harvested at indicated times; (**B**) Shows the quantification of the same from three independent experiments with standard error of mean (**C**) Western blot indicating requirement of active HCMV replication for TNKS accumulation. HFFs were infected with HCMV Towne or UV-inactivated HCMV Towne at MOI = 1 and harvested at 72 hpi; (**D**) Shows the quantification of the same from three independent experiments with standard error of mean (**E**) qRT-PCR showing mRNA levels of TNKS 1 and 2, Axin 1 and β-catenin at 24 hpi in infected HFFs. Quantification is averaged from four independent experiments with standard error of means. * indicates *p* value < 0.05, ** indicates *p* < 0.01, and *** indicates *p* < 0.001 and ns indicates non-significance.

HCMV infection is characterized by an increase in Axin1 expression followed by degradation of β-catenin [13,14], while infection with HSV1 or HSV2 does not result in β-catenin degradation [15]. The mechanism(s) involved in Axin1 accumulation by HCMV remain to be determined. Axin1 expression is tightly regulated in the cell; one mechanism of its regulation is through PolyADP Ribose Polymerase 5 (PARP5), also known as TNKS [12]. To determine whether the reported Axin1 accumulation in HCMV is mediated by TNKS, the effect of HCMV infection on the expression of TNKS 1 and 2, Axin1 and β-catenin was measured in HFFs at 24, 48 and 72 hours post infection (hpi). This was compared to HSV1-infected HFFs which were harvested at 6, 24 and 48 hpi. TNKS has two isoforms, 1 and 2, and the antibody used in our study can identify both and, henceforth, is referred to as TNKS. At all time points, Axin1 was induced and β-catenin was decreased in HCMV-infected HFFs, in agreement with previous reports [13,14]. There was a distinct accumulation of TNKS at all time points (Figure 1A and quantified in Figure 1B) accompanying the Axin1 accumulation. In contrast to HCMV, HSV1 did not significantly modify the levels of TNKS, Axin1, or β-catenin, in agreement with a previous report [15]. These results suggest that the observed changes in TNKS and Axin1 might be HCMV-specific.

To examine whether HCMV replication was responsible for the accumulation of TNKS, HFFs were infected with UV-inactivated HCMV and lysates prepared from these cells were compared to lysates from wild-type HCMV-infected HFFs. Ultraviolet (UV)-inactivation of HCMV was confirmed by the absence of IE1/IE2 expression (Figure 1C). TNKS accumulation was not observed in cells infected with the UV-inactivated Towne (Figure 1C and quantified in Figure 1D), indicating that active HCMV replication was required for TNKS accumulation.

### 2.2. Effect of HCMV Infection on TNK Accumulation is Post-Translational

**Figure 2:**
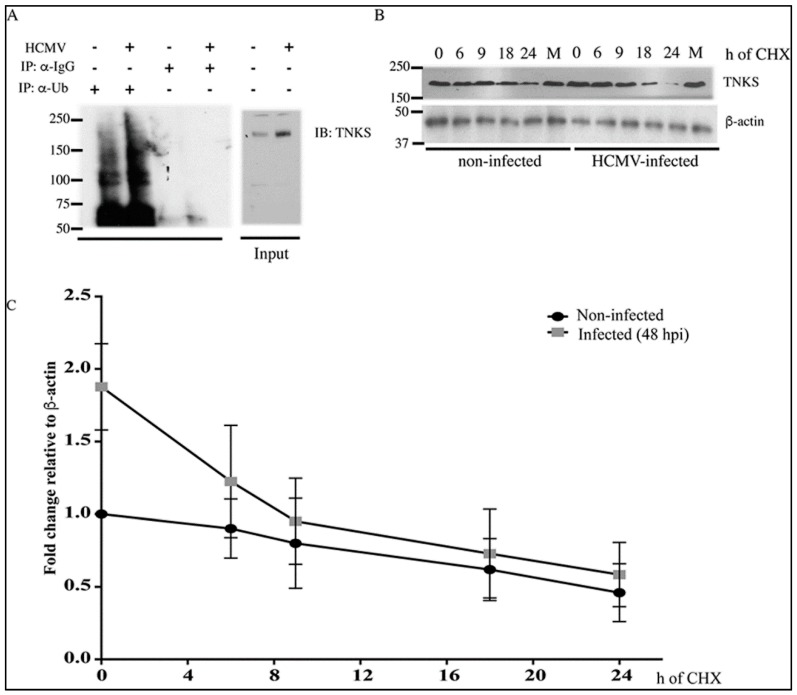
TNKS degradation is not inhibited upon HCMV infection. (**A**) Western blot showing ubiquitination of TNKS immunoprecipitated from non-infected and HCMV-infected HFFs using an anti-ubiquitin antibody. HFFs were infected with HCMV Towne at MOI = 1 and harvested at 72 hpi; (**B**) Western blot showing stability of TNKS after 48 h of infection at MOI = 3 followed by treatment with cycloheximide to inhibit *de novo* translation and harvested at indicated time points. M indicates treatment with MG132 as a proteasome inhibitor control; (**C**) Quantification of TNKS’ degradation normalized to β-actin from (**B**) Cycloheximide was added at 48 hpi for the times indicated in the X axis (h of CHX). Data shown are the average of three experiments with standard error of the means.

HCMV upregulates multiple host proteins by inducing mRNA synthesis through viral transcription factors, and TNKS might be upregulated in a similar way. However, qRT-PCR at 24 hpi revealed that mRNA levels of TNKS 1 and 2 were not significantly changed during infection, indicating the upregulation of TNKS was a post-transcriptional effect (Figure 1E). Additionally, Axin1 or β-catenin were not induced at the mRNA level, indicating that the Wnt pathway is likely modulated at the protein level, in agreement with [13]. Accumulation of TNKS could result from decreased protein ubiquitination and subsequent reduction in its proteasomal degradation [12,16]. TNKS ubiquitination was measured by immunoprecipitation (IP) using anti-ubiquitin antibody and immunoblot using TNKS antibody (Figure 2A). A significant and reproducible difference in the level of ubiquitinated TNKS between non-infected and HCMV-infected human foreskin fibroblasts (HFFs) was observed, suggesting that the ubiquitination status of TNKS was increased, rather than decreased upon infection (Figure 2A). These results were also supported by the degradation of TNKS, measured in the presence of cycloheximide, a translational inhibitor, in non-infected and HCMV-infected HFFs after 48 hpi (Figure 2B and quantified in Figure 2C). TNKS degradation occurred in both non-infected and HCMV-infected lysates, indicating that proteasomal degradation of TNKS was not inhibited by HCMV. Taken together, TNKS accumulation in HCMV-infected cells was not due to an upregulation of transcription or post-translational changes resulting in altered degradation.

### 2.3. HCMV Infection Reduces PARsylation Activity of TNKS

**Figure 3:**
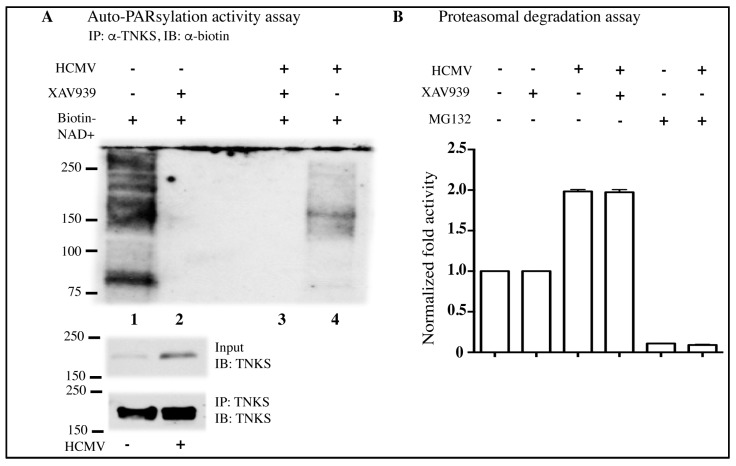

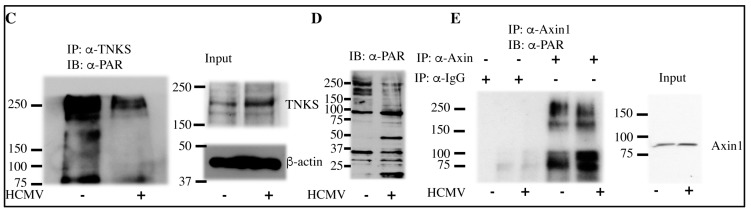
HCMV inactivates TNKS activity. (**A**) Western blot showing auto-PARsylation activity of immunoprecipitated TNKS at 72 hpi with HCMV Towne at MOI = 3. Immunoprecipitated TNKS was used for the PARsylation assay as described in Materials and Methods. XAV939 was included as a positive control for TNKS inhibition; (**B**) Proteasomal degradation assay from lysates assayed in (**A**) to indicate lysate quality and activity; (**C**) Western blot showing detection of PAR residues on TNKS immunoprecipitated from non-infected and HCMV-infected HFFs (MOI = 3 at 72 hpi); (**D**) Detection of PAR residues by Western blot in non-infected and HCMV Towne-infected HFF (MOI = 3, 72 hpi) cell lysates to show no loss of PARsylation activity from other PARPs in general; (**E**) Western blot showing detection of PAR residues on Axin1 immunoprecipitated from non-infected and HFFs infected by HCMV Towne at MOI = 3 at 72 hpi.

Since transcriptional change or protein degradation did not seem to play a role in TNKS accumulation, we hypothesized that HCMV infection could influence the enzymatic activity of TNKS. Inhibition of TNKS activity can lead to its accumulation, and inhibition of auto-PARsylation of TNKS by XAV939 (a chemical inhibitor of TNKS) results in TNKS accumulation in multiple cell systems [12,17,18]. The contribution of auto-PARsylation activity to TNK accumulation was therefore tested in infected cells using an *in vitro* semi-quantitative biochemical assay. Following IP of TNKS, its auto-PARsylation activity was determined by measuring its ability to use biotin-labeled nicotinamide adenine dinucleotide (NAD^+^) as a substrate and catalyze the addition of biotin-labeled ADP to itself. The biotin label on TNKS was measured by Western blot. TNKS from non-infected HFFs exhibited auto-PARsylation activity, evident by detection of biotin bound to TNKS, but in HCMV-infected HFFs, there was a consistent and reproducible reduction in the biotin-ADP signal detected on TNKS (Figure 3A). As expected, XAV939 inhibited the auto-PARsylation activity of TNKS when included in the PARsylation reaction (Figure 3A). The lysates from non-infected and infected cells used in the TNKS assay exhibited the expected activity when assayed for proteasome-mediated degradation (Figure 3B), indicating that the quality of extracts was not compromised. In addition, the general proteasomal activity was unchanged in extracts treated with XAV939, as expected. The TNKS activity assay indicated that HCMV directly reduced the auto-PARsylation activity of TNKS1/2. The differences observed in auto-PARsylation activity were expected to result in changes in the abundance of PAR residues on TNKS in these lysates. An antibody directed against PAR residues detected a weaker signal on TNKS immunoprecipitated from HCMV-infected cells as compared to non-infected cells (Figure 3C). Thus, HCMV-infected cells had reduced TNKS activity that resulted in reduced TNKS auto-PARsylation. The reduction in TNKS-mediated PARsylation was likely specific, since general PARsylation was not reduced when whole-cell lysates were probed with the anti-PAR antibody (Figure 3D). Thus, HCMV infection does not reduce protein PARsylation in general, but specifically inhibits the PARsylation activity of TNKS.

Axin1 is a target protein for TNKS-mediated PARsylation. The reduction in TNKS activity in HCMV-infected cells was accompanied by a decrease in PARsylation of Axin1 immunoprecipitated from infected cells (Figure 3E) when compared to non-infected cells. TNKS has only two binding motifs on Axin1 [19]; therefore, detection of PARsylation on Axin1 is more difficult than detection of TNKS auto-PARsylation. Thus, HCMV-mediated reduction in TNKS activity translated into reduced PARsylation of both TNKS and Axin1, which may contribute to increased stability and accumulation of both proteins in infected cells.

### 2.4. Inhibition of TNKS Activity Promotes HCMV Replication

**Figure 4:**
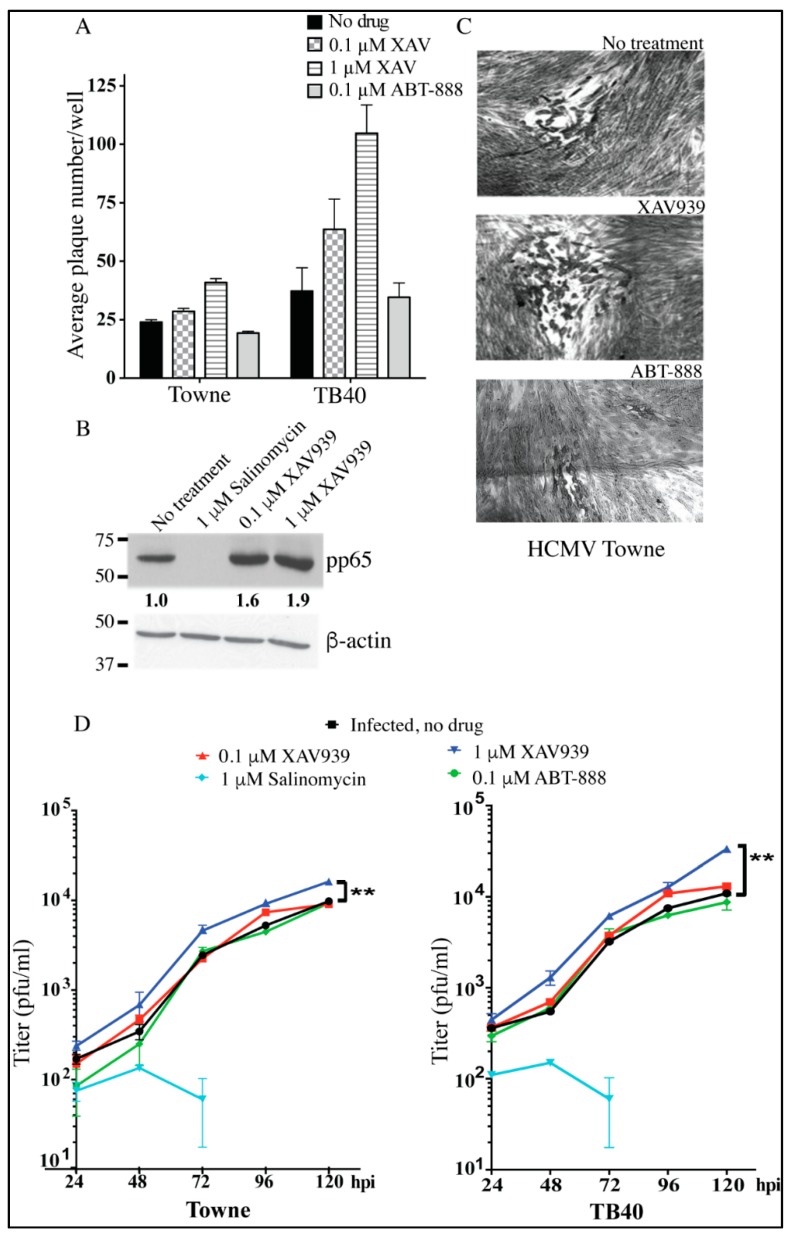
TNKS inhibition favors HCMV replication. (**A**) Plaque assay for HCMV Towne and TB40 strains with XAV939 (PARP5 inhibitor), and ABT-888 (PARP1and 2 inhibitor). HFFs were infected to yield approximately 50-100 plaques/well, stained and quantified at 10 days after plating; (**B**) Western blot to detect viral proteins at 72 hpi in HFFs infected with HCMV Towne at MOI = 1. Fold change in protein expression normalized to β-actin are quantified under the blot; (**C**) Plaques from (**A**) imaged after staining at 10× magnification; (**D**) Viral growth assay for Towne and TB40 strains with XAV939 (PARP5 inhibitor), Salinomycin (Wnt inhibitor) and ABT-888 (PARP1and 2 inhibitor). HFFs were infected and supernatants were collected at 24-h intervals till 120 hpi. Released virions were titered by a standard plaque assay. Average pfu/mL calculated from quadruplicate wells of one experiment are shown. * indicates *p* value < 0.05, ** indicates *p* < 0.01.

Since HCMV inhibited the PARsylation activity of TNKS, the effect of chemical inhibition of TNKS’ activity on HCMV replication was studied. Compounds that inhibit the Wnt pathway were reported as HCMV inhibitors [14]; the Wnt inhibitor, Salinomycin, reduced Wnt signaling and suppressed HCMV replication (6). XAV939, an enzymatic inhibitor of TNKS, inhibits the Wnt pathway by upregulating Axin1 [12]. We used XAV939 in HCMV-infected HFFs at concentrations that inhibit TNKS activity (0.1 and 1 μM) [20,21]. At these concentrations, XAV939 did not change PARP1 or PARP2 at the mRNA level (Figure S1). Unlike the reported Wnt inhibitor, Salinomycin, XAV939 did not inhibit HCMV replication (Figure 4A,B). A plaque reduction assay performed with the laboratory-adapted strain of HCMV, Towne, and the TB40 endotheliotropic strain showed a slight increase in both the number and size of plaques (Figure 4A,C and Figure S2) with XAV939 treatment, suggesting that TNKS inactivation may confer a favorable environment for HCMV replication (Figure 4A). Unlike the strong inhibition of HCMV-encoded pp65 expression by Salinomycin, no inhibition was observed with XAV939 (Figure 4B). To test whether inhibition of TNKS (PARP5) reflects general PARP inhibition by HCMV, the PARP1and 2 inhibitor, ABT-888, was also used. There was a significant reduction in plaque size with ABT-888 (Figure 4C and Figure S2), but the number of infectious foci was not significantly reduced (Figure 4A). A growth assay (Figure 4D) similarly showed that XAV939 improved HCMV growth, while Salinomycin fully inhibited HCMV. ABT888 did not affect the number of virions. These results suggest that HCMV-mediated inhibition of PARP activity could be specific to PARP5. Other PARPs may still play a role in virus propagation. Taken together, inhibition of TNKS activity alone creates a favorable environment for HCMV replication and inhibition of TNKS activity might be a strategy that HCMV exploits for a variety of cellular effects.

**Figure 5:**
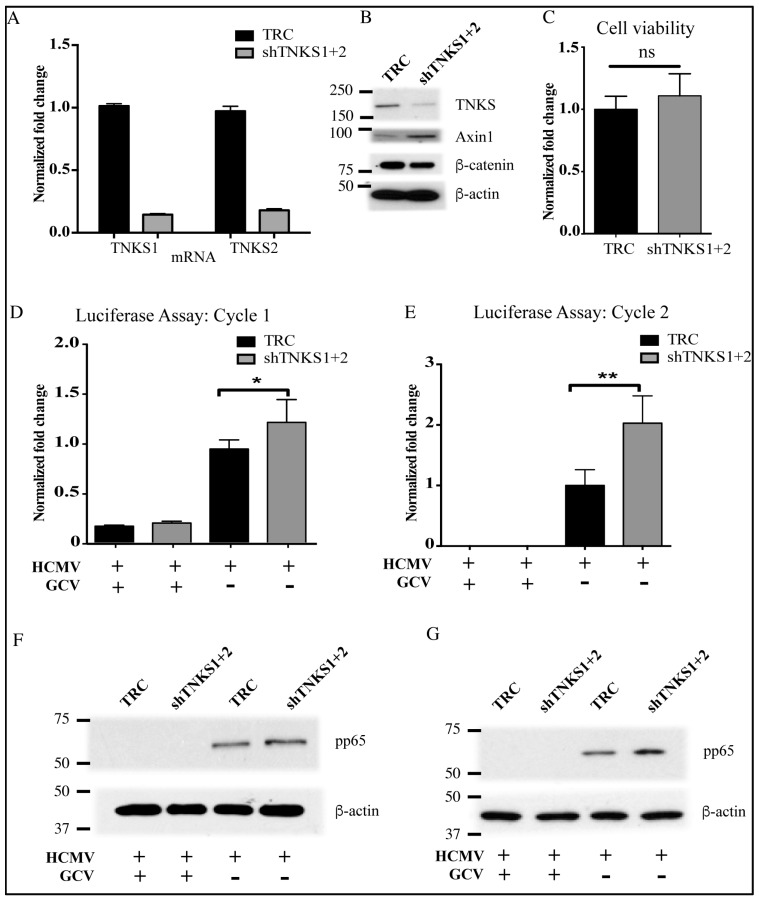
TNKS knockdown improves HCMV replication in HFFs. (**A**) qRT-PCR showing reduction in TNKS1 and TNKS2 mRNA level in double knockdown cells compared to control (TRC); (**B**) Western blot showing effect of TNKS knockdown on expression of Wnt proteins (TNKS, Axin1 and β-catenin); (**C**) Cell viability assay for control and TNKS knockdown lines; (**D**) pp28-luciferase assay to measure viral growth in control and TNKS knockdown cell lines; (**E**) Luciferase and Western blot (**F**) of second cycle infection from D; (**G**) Western blot to detect viral protein pp65 in lysates from (**E**) * indicates *p* value < 0.05, ** indicates *p* < 0.01, and ns indicates non-significance.

To further confirm the role of TNKS during HCMV infection, TNKS1 and TNKS2 were knocked down in HFF using shRNAs. TNKS knockdown reduced levels of both TNKS1 and TNKS2 mRNA (Figure 5A) and protein (Figure 5B). Reduction of TNKS levels increased endogenous Axin1 and reduced β-catenin in HFFs (Figure 5B) as expected [12]. Knockdown of TNKS did not alter cell viability or change cell growth when compared to the control cells (Figure 5C). Growth of a pp28-luciferase Towne strain was improved in the TNKS knockdown cell line when compared to the control line, both by luciferase counts (Figure 5D) and pp65 expression (Figure 5F). Ganciclovir (GCV) inhibited virus replication in both cell lines. Virion release from the two lines was also measured in second cycle assay using supernatants from Figure 5D, showing more virions were released from the TNKS knockdown cell line as compared to the control line by pp28-luciferase activity (Figure 5E) and pp65 expression (Figure 5G). Thus, knockdown of TNKS conferred a growth advantage for HCMV.

### 2.5. Accumulation of TNKS during Infection Correlates with Wnt Inhibition

**Figure 6:**
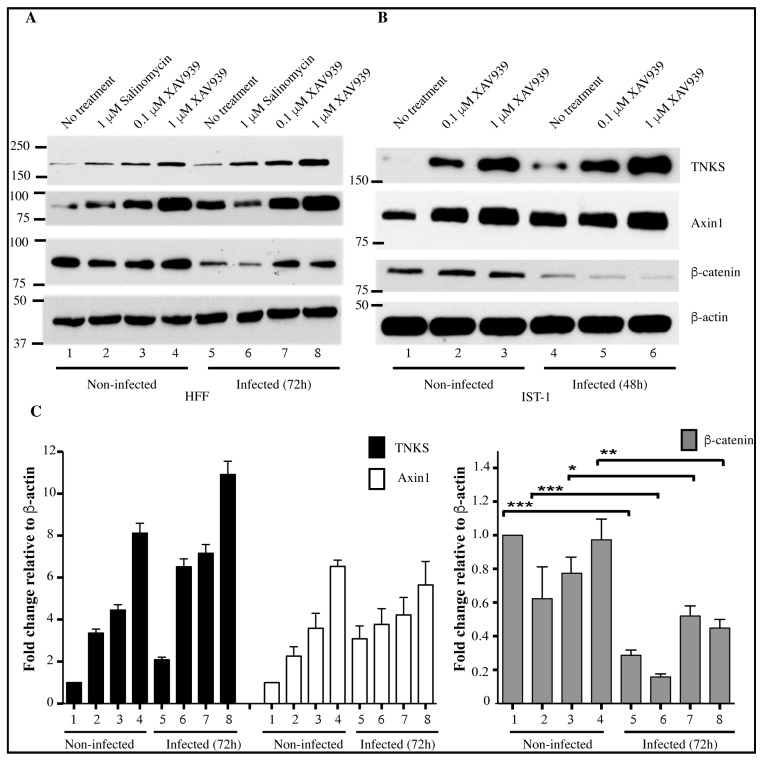

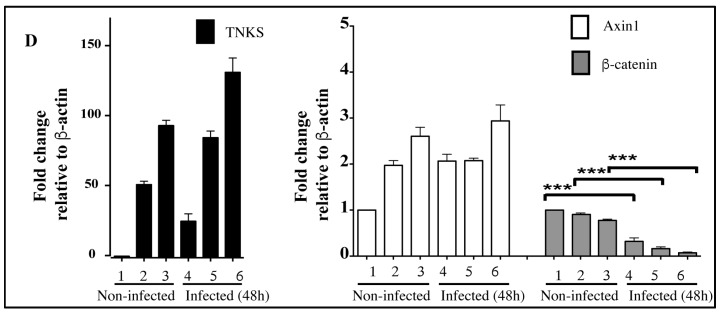
HCMV-induced TNKS accumulation correlates with decreased β-catenin. Western blot showing levels of Wnt proteins (TNKS, Axin1, β-catenin) in infected HFFs (**A**) and IST-1 (**B**) cells. XAV939 (0.1 and 1 μM) and Salinomycin (0.1 μM) were used as modulators of Wnt. TNKS accumulates in response to HCMV infection and β-catenin is reduced upon infection; (**C**) and (**D**) show the quantification of (**A**) and (**B**) respectively from three representative experiments. Data plotted is mean ± standard error, SE. Lane numbers in Western blot figures correspond to lane numbers in quantification plots. * indicates *p* value < 0.05, ** indicates *p* < 0.01, and *** indicates *p* < 0.001, for changes in β-catenin.

Since downregulation of TNKS activity was an HCMV-driven mechanism, we investigated the effects of TNKS inhibition on the expression of Wnt pathway proteins during HCMV infection. Changes in TNKS, Axin1 and β-catenin expression were measured in HFFs and the placental cytotrophoblastic cell line, IST-1 (Figure 6A,B, quantified in Figure 6C,D), treated with XAV939 or Salinomycin. In both cell lines, the induction of Axin1 by HCMV resulted in reduced expression of β-catenin (Figure 6A, lanes 1 and 5, and Figure 6B, lanes 1 and 4). Inactivation of TNKS by XAV939 is expected to lead to its cellular accumulation. This was clearly observed by the dose-dependent increase of TNKS levels with XAV939 (Figure 6A lanes 3–4 in HFFs, Figure 6B, lanes 2–3 in IST-1). Whenever XAV939 induced accumulation of inactive TNKS, HCMV was able to drive even further accumulation of TNKS (Figure 6A, lanes 7–8, compared to lanes 3–4, and Figure 6B, lanes 5–6, compared to lanes 2–3). An increase of TNKS levels was accompanied by accumulation of Axin1 (Figure 6A, lanes 3–4, and Figure 6B, lanes 2–3), in agreement with the expected effect of TNKS inhibition on Axin1 accumulation. In all instances, infection with HCMV resulted in reduced β-catenin levels, compared to the levels observed in non-infected cells (Figure 6A, lanes 7–8, compared to lanes 3–4, and Figure 6B, lanes 5–6, compared to lanes 2–3). Infection resulted in reduced levels of β-catenin, irrespective of the amount of Axin or TNKS. Data in Figure 6C,D indicate that HCMV always increases TNKS and Axin1 while lowering β-catenin, irrespective of the starting basal level of these proteins. Therefore, a robust HCMV-driven mechanism is in place for TNKS inactivation, which plays a role in inhibition of the Wnt/β-catenin pathway. However, HFFs and intermediate trophoblastic cell line, ISTs were not as sensitive to XAV939-mediated suppression of β-catenin (Figure 6A, lanes 3–4 and Figure 6B, lanes 2–3) as has been reported in colon cancer cell lines [12]. This “non-responsiveness” to XAV939 has also been reported in other cancer cell-lines [22]. In the colon cancer cell line SW480, we also found that β-catenin was reduced in the presence of XAV939 (Figure S3). Thus, although use of XAV939 sheds light on the requirement for inactivating TNKS for HCMV infection, HCMV-mediated control of β-catenin may involve additional Wnt proteins that are not directly controlled by TNKS.

## 3. Discussion

HCMV often manipulates host cell proteins to promote its replication. There are multiple instances of HCMV co-opting critical cellular pathways to assist its replication, including cell cycle regulators and other signaling pathways. HCMV recruits pRb, Cyclin Dependent Kinases (CDKs) [23], p53 [24,25], Pro Myelocytic Leukemia (PML) [25] and other cellular proteins for efficient replication and at various points during its replication [26].

We reported previously that HCMV upregulated Axin1 and reduced β-catenin [14]. Inhibitors of the Wnt pathway such as Salinomycin and Monensin efficiently suppressed HCMV replication, suggesting a fine balance of the Wnt pathway is important for HCMV replication. In the current study, we show that HCMV inactivates TNKS, the negative regulator of Axin1. Infection leads to accumulation and stabilization of TNKS in infected cells resulting from its enzymatic inhibition and reduced PARsylation activity. Reduced PARsylation may also explain the changes in Axin1 stability. Although the direct mechanism by which HCMV inactivates TNKS activity remains to be elucidated, it is possible that viral or even cellular proteins induced by infection interact with TNKS and slow down its enzymatic activity. A protein array targeting cellular proteins that interact with HCMV Immediate Early 1 (IE1) identified Tankyrase1 Binding Protein 1 (TNKS1BP1) as an IE1-interacting protein based on hybridization and IP [27], supporting the requirement for active virus replication and *de novo* synthesis of HCMV proteins (particularly IE1) for TNKS accumulation. Thus, it is possible that viral components target TNKS and regulate its activity. In a recent study [28] using a genome-wide deep sequencing and ribosome profiling approach, no significant increase in TNKS translation with HCMV infection at 5–72 h of infection was seen. Thus, TNKS stabilization may be controlled by post-translational modification. In addition, rates of protein production and turnover may change under different cellular conditions [29].

Using a semi-quantitative assay, we observed a decrease in the auto-PARsylation activity of TNKS upon infection, which should be expected to reduce its degradation. However, TNKS was degraded efficiently in both non-infected and HCMV-infected HFFs. This apparent conflict probably is due to residual PARsylation activity in HCMV-infected HFFs. Thus, unPARsylated TNKS might accumulate slowly, but eventually TNKS will get PARsylated, ubiquitinated and degraded even in infected HFFs. HCMV slows down the PARsylation activity of TNKS, which is reflected in its accumulation during infection. The accumulated TNKS plays an as yet undefined role in HCMV replication. Reduction in TNKS activity in infected cells is also reflected in some reduction in Axin1 PARsylation in infected cells. However, TNKS-mediated regulation may not be the only mechanism leading to Axin1 stabilization in HCMV infection. Axin1 levels are tightly controlled by the “destruction complex” (DC) present in the cytoplasm. KSHV employs a mechanism targeting the critical DC protein GSK-3β that involves LANA-mediated redistribution of GSK-3β [30]. Similarly, the involvement of APC and casein kinase 1 (CKI) in HCMV-mediated Wnt modulation remains to be explored, and might still play a significant role in regulating Axin1 stability and degradation during HCMV infection.

Studies of the interdependence of TNKS and Axin and how they mutually regulate each other identified RNF146 (RING Finger Protein), an ubiquitin E3 ligase which serves as a positive regulator of Wnt signaling [17,31]. RNF146, TNKS, and Axin formed a protein complex, and RNF146 was responsible for ubiquitination of the trimer and targeting it for proteasomal degradation. A Trp–Trp–Glu (WWE) motif in RNF146 recognized PARsylated proteins for degradation through a direct binding between the WWE domain and PAR moiety. In addition to TNKS, other target proteins are modified by the same mechanism. TNKS activity reduced RNF146 protein levels. The mechanism of Wnt regulation via RNF146 is entirely unexplored in HCMV and it is possible that HCMV might modify or change the equilibrium of the proteins in the trimer, thus modulating changes in Wnt.

The WWE domain of RNF146 shown to be a PAR-binding domain is also found in other E3 ligases, namely, HUWE1 (HECT, UBA and WWE domain containing 1), DTX1–4 (deltex homologues 1–4), TRIP12 (thyroid hormone receptor interactor 12), reviewed in [32]. These E3 ligases may also regulate protein turnover of TNKS and Axin in a PARsylation-dependent manner. It is possible that HCMV co-opts this finely-tuned mechanism for protein degradation in the cell.

The accumulation of TNKS during HCMV infection and resulting Axin1 stabilization and decrease in β-catenin expression is a unique feature of HCMV infection. The effect of XAV939 on β-catenin expression may be both time- and cell-dependent, since in HFFs, despite the accumulation of TNKS and Axin1, the expression of β-catenin was determined largely by infection and not by XAV939. Initial studies of XAV939 in the colon carcinoma in SW480 cells revealed that exposure to XAV939 resulted in reduced levels of β-catenin. Similarly, we found the same effect of XAV939 on β-catenin in SW480 cells, which differed from its effects in HFFs (Figure S3). Reports suggest that the outcome of XAV939 treatment may be cell-type specific, and, in some cells, prolonged exposure (>6 h) to XAV939 results in a reduced effect on β-catenin inhibition [22]. XAV939 insensitivity was conferred by β-catenin’s association with Lymphoid-enhancer factor 1 (LEF1) and B cell lymphoma 9 (BCL9-2/B9L), which normally accumulate during Wnt stimulation, suggesting that β-catenin inhibition requires the targeting of its interaction with LEF1 and/or BCL9/B9L. Alternatively, it is possible that mechanisms other than the TNKS-Axin axis are involved in β-catenin inhibition by HCMV.

## 4. Materials and Methods

### 4.1. Compounds

XAV939, an inhibitor of TNKS activity, Salinomycin, an inhibitor of the Wnt pathway that decreases the phosphorylation of low-density lipoprotein receptor-related protein 6, and ABT-888, PARP1 and PARP2 inhibitor, were obtained from Sigma-Aldrich (St. Louis, MO, USA) and dissolved in water or DMSO. A stock of 10 mM was stored at −80 °C. The 50% inhibitory concentration (IC_50_) of XAV939 for TNKS1 and 2 is 10 and 4 nM, respectively and for PARP1and 2–2.2 and 0.1 μM. Therefore, XAV939 was used at concentrations of 0.1 and 1 μM, which are specific for inhibition of TNKS1 and 2. The IC50 of ABT88 towards PARP1 and TNK1 is 5.2 nM, and 15 μM; therefore, it was used at 0.1 μM. Cycloheximide (Sigma-Aldrich) was dissolved in DMSO and used at 100 μg/mL. Ganciclovir (Sigma-Aldrich) was dissolved in PBS and used at 5 μM.

### 4.2. Viruses

The HCMV strains, Towne (American Type Culture Collection, ATCC, Manassas, VA, USA, VR-977) and TB40-GFP (ATCC VR-1578) were used as specified for each experiment. Towne HCMV was UV-inactivated by spreading a thin layer of stock suspension in an uncovered six-well tissue culture plate and exposing to a total dose of 720 mJ/cm^2^ in a UV crosslinker (Spectrolinker XL-1000, Spectronics, Westbury, NY, USA) [33]. A clinical isolate of HSV1 was obtained from the clinical microbiology laboratory at Johns Hopkins, with no identifiers that could be linked to a patient. The pp28-luciferase Towne strain was constructed as previously described [34]. This recombinant virus expresses luciferase under the control of the UL99 (pp28) late promoter 48–72 h post infection (hpi) and luciferase activity highly correlates with plaque reduction assay.

### 4.3. Cell Culture, Virus Infection and Anti-Viral Assays

Human foreskin fibroblasts (HFFs) passage 12–16 (ATCC, CRL-2088) were grown in Dulbecco’s Modified Eagle Medium (DMEM) (Life Technologies, Grand Island, NE, USA) containing 10% fetal bovine serum (FBS) (Gibco, Carlsbad, CA, USA) in a 5% CO2 incubator at 37 °C and used for infection with HCMV. The cytotrophoblastic cell line, IST-1, was provided by Ie Ming-Shih, Johns Hopkins University School of Medicine, and was maintained in RPMI 1640 (Life Technologies, Grand Island, NY, USA) containing 10% FBS in a 5% CO2 incubator at 37 °C [35]. Colorectal adenocarcinoma, SW480 cells (ATCC CCL-228) were grown in L-15 medium containing 10% FBS (Gibco) under atmospheric conditions at 37 °C. When used, MG132 (10 μM) was included in the culture medium for 8–10 h before harvesting. Cell viability was tested using the CellTiter-Glo luminescent assay kit (Promega, Madison, WI, USA) according to manufacturer’s instructions.

Infection was carried out at multiplicity of 1 plaque forming unit (PFU)/cell (MOI = 1), unless otherwise noted. Following 90 min adsorption, media containing virus was removed and replaced by DMEM with 4% FBS (Gibco) with or without compounds. Infected or infected-treated HFFs were collected at specific time points depending on the assay used. For plaque reduction assay, HFFs were seeded at 1 × 10^6^ cells/ 24-well plate one day prior to infection. HCMV (Towne or TB40) was diluted in DMEM to a desired titer which gave approximately 50 plaques/well and added to each well in quadruplicates. Plates were incubated for 90 min with shaking every 10 min; thereafter overlaid with carboxy methyl cellulose supplemented with XAV939 or ABT-888. After 10 days of incubation, cells were stained with crystal violet and plaques were counted at 40× magnification and photographed using a Nikon Eclipse E-800 fluorescence microscope (Nikon, Melville, NY, USA).

The pp28-luciferase Towne virus was used for estimation of viral growth. Infected HFFs were collected at 96 hpi, and pp28 activity was measured by a luciferase assay kit (Promega) on the GloMax-Multi detection system (Promega). In second-cycle replication assays, supernatants were collected from the tested conditions of the first cycle and used for infection of fresh HFFs in 96-well plates. Luciferase activity was measured 120 h after second-cycle infection.

Viral growth assay was carried out as reported [34,36]. Briefly, HFFs were infected with HCMV Towne or TB40 (MOI 0.1) and treated with drugs. Culture supernatants were collected every 24 h until 120 hpi and frozen at −80 °C. Collected samples were thawed and used for titration of infectious virus by plaque assay.

### 4.4. SDS-PAGE and Immunoblot Analysis

Cell lysates were quantified for total protein content using Pierce bicinchoninic acid (BCA) protein assay kit (Thermo-Fisher Scientific, Waltham, MA, USA). Equivalent amount of proteins were mixed with an equal volume of sample buffer (125 mM Tris-HCl (pH 6.8), 4% SDS, 20% glycerol, 5% β-mercaptoethanol) and boiled at 100 °C for 10 min. Denatured proteins were resolved in Tris-glycine polyacrylamide gels (10%–12%) and transferred to polyvinylidene difluoride membranes (Bio-Rad Laboratories, Hercules, CA, USA) by electroblotting. Membranes were incubated in blocking solution (5% nonfat dry milk/BSA and 0.1% Tween 20 in PBS (PBST)) for 1 h, washed three times with PBST, and incubated with primary antibodies diluted in 5% milk at 4 °C overnight. The membranes were washed with PBST, followed by incubation with horseradish peroxidase (HRP)-conjugated secondary antibodies in 5% milk/BSA for 1 h at room temperature. After three washing steps with PBST, protein bands were visualized by chemiluminescence using SuperSignal West Pico reagent (Pierce Chemical, Rockford, IL, USA). The following antibodies were used: mouse monoclonal anti-pp65 antibody (Vector Laboratories, Burlingame, CA, USA), mouse monoclonal anti-PAR antibody (Trevigen, Gaithersburg, MD, USA), rabbit monoclonal anti-Axin1 (Cell Signaling Technologies, Danvers, MA, USA), rabbit polyclonal anti-β-catenin, anti-TNKS 1 and 2 (Santa Cruz Biotechnologies, Santa Cruz, CA, USA), mouse monoclonal anti-ubiquitin and anti-β-actin antibody (Santa Cruz Biotechnology), HRP-conjugated goat anti-rabbit IgG antibody (Cell Signaling Technology), and HRP-conjugated sheep anti-mouse IgG (GE Healthcare, Waukesha, WI, USA). Protein A-HRP was obtained from ThermoFisher Scientific (Grand Island, NY, USA). Proteins levels were quantified using ImageJ 1.48v (NIH, Bethesda, MD, USA).

### 4.5. Protein Immunoprecipitation (IP)

Approximately 10^7^ HFFs were lysed with RIPA buffer and total lysate was precleared with 50 μL protein A/G agarose bead slurry (Santa Cruz) for 1 h. The precleared lysates were quantified as described above and 500 μg to 1 mg of total lysate was incubated overnight with anti-ubiquitin (1 μg), anti-TNKS (2 μg), or anti-Axin1 (2 μg) antibody. The antibody–lysate complexes were incubated with protein A/G beads (Santa Cruz) at 4 °C and washed three times with RIPA buffer. Samples were boiled in SDS sample buffer, and the supernatant was analyzed on SDS-PAGE gel after immunoblotting as described previously. Then, 1%–5% of the cell lysate used for IP was loaded on gels as “Input”.

### 4.6. IP of TNKS and *In Vitro* PARsylation Assay

The protocol was modified from [12] and [37]. Briefly, 10^7^ HFFs were pelleted and resuspended in 1 mL TNE buffer (10 mM Tris (pH 8.0), 1% Nonidet P-40, 0.15 M NaCl, 1 mM EDTA) for 1 h at 4 °C. Cells were lysed by passing 6 times through 25 5/8 gauge syringe, and supernatant was collected after centrifugation at 14,000 rpm for 10 min. The lysate was pre-cleared with 50 μL Protein A/G beads (Santa Cruz) for 2 h at 4 °C, and approximately half of the lysate was used per reaction. TNKS was immunoprecipitated using 1 μg TNKS1/2 antibody per mL TNE extract for 5 h at 4 °C. The antibody-lysate mix (500 μL) was loaded on 25 μL protein A/G beads per reaction (pre-washed with TNE buffer (0.5–1mL/wash, 2 washes)) for an overnight incubation at 4 °C. Beads were washed thrice with TNE and twice with 50 mM Tris-HCl (8.0). TNKS assay buffer ((50 mM Tris-HCl (pH 8.0), 4 mM MgCl_2_, 0.2 mM dithiothreitol (DTT)) was then added with Biotin NAD^+^ (5 μM, Trevigen) with/without XAV939 (1–5 μM, final concentration). The reaction was incubated at 30 °C for 2.5 h, the beads were boiled after adding 5 μL 5× SDS gel loading buffer (Bromophenol blue (0.25%), DTT (dithiothreitol; 0.5 M), Glycerol (50%), SDS (sodium dodecyl sulfate; 10%), Tris-HCl (0.25 M, pH 6.8) to the reaction. The samples were subjected to PAGE on 8% gel and blocked with 5% BSA, and incubated with streptavidin HRP (Pierce) at 1:20,000 to 5% BSA for overnight at 4 °C. The blot was washed with PBST and detected as described above.

### 4.7. Determination of TNKS Degradation

For estimation of TNKS degradation, non-infected and HCMV-infected HFFs (48 hpi) were treated with cycloheximide (100 μg/mL) diluted in DMEM with 4% FBS and harvested at 3 h intervals for 3 h through 24 h for SDS-PAGE analysis as described above.

### 4.8. Proteasome Assay

Proteasome activity was determined as reported [38]. Briefly, non-infected and infected HFFs (MOI = 3, 72 hpi) were harvested and lysed in Proteasome Lysis/Assay Buffer containing 50 mM HEPES (pH 7.8), 10 mM NaCl, 1.5 mM MgCl_2_, 1 mM EDTA, 1 mM EGTA, 250 mM sucrose, and 5 mM DTT. 50 μL of cell lysate was incubated with 100 μM Suc-LLVY-AMC (chymotrypsin-like activity substrate) (Enzo Life Sciences, Farmingdale, NY, USA) diluted in 200 μL of proteasome assay buffer (lysis buffer supplemented with 2 mM ATP) and incubated at 37 °C for 60 min. The released fluorogenic AMC was measured at 360 nm excitation and 460 nm emission using a fluorometric plate reader (GloMax, Promega) and normalized to protein amount determined by standard BCA-based protein estimation assay (Pierce). MG132 (1 μM) was included in samples as a control for proteasome activity.

### 4.9. RNA Isolation and Real-Time Quantitative Reverse Transcriptase (qRT) PCR

Total RNA was isolated from non-infected and HCMV-infected HFFs (with and without drug treatment) using RNeasy Mini kit (Qiagen, Georgetown, MD, USA) according to manufacturer’s instructions. RevertAid first strand cDNA synthesis kit (Life Technologies, Carlsbad, CA, USA) was used to synthesize first strand cDNA from 2 µg total RNA using oligo-dT primers. Negative reverse-transcriptase (-RT) reactions were included to ensure the specificity of qRT-PCR reactions. Synthesis of first strand cDNA from mRNA template was carried out at 42 °C for 1 h. cDNAs were diluted by 5 fold and quantitative RT-PCR (qRT-PCR) was performed using 2 µL of diluted cDNA, specific primers and SYBR green (Life Technologies) with two-step cycling protocol (95 °C for 15 s, 55 °C for 1 min). Reactions were performed in triplicates and GAPDH was used as internal control. mRNA levels in HCMV-infected cells were normalized to the mRNA produced in non-infected HFFs in addition to the internal normalization of each sample to GAPDH. Primers used to detect the mRNAs were: 5′-GTCCCTGACAGCCTAGAAATAAG-3′ and 5′-GGGAACAGTAGCAGTTGAGTATG-3′ for TNKS1, 5′-TCCGGACAACAAGGTCTTAAC-3′ and 5′-CTCTTCCTCCACAGACTGAAAC-3′ for TNKS2, 5′-CTTCACCTGACAGATCCAAGTC-3′ and 5′-CCTTCCATCCCTTCCTGTTTAG-3′ for β-catenin, 5′-GAGGTATGTGCAGGAGGTTATG-3′ and 5′-TCCTCTGCGATCTTGTCTCT-3′ for Axin1, 5′-GCCGAGATCATCAGGAAGTATG-3′ and 5′-ATTCGCCTTCACGCTCTATC-3′ for PARP1, 5′-GTGGAGAAGGATGGTGAGAAAG-3′ and 5′-CTCAAGATTCCCACCCAGTTAC-3′ for PARP2 and 5′- TTGGTATCGTGGAAGGACTC-3′ and 5′-ACAGTCTTCTGGGTGGCAGT-3′ for GAPDH.

### 4.10. Knockdown of TNKS

ShRNA clones targeting TNKS2 (TRCN0000053239, TRCN0000053240, TRCN0000053241, TRCN0000053242, TRCN0000053238) and TNKS1 (TRCN0000040187 and TRCN0000040185) were obtained from Sigma. To package lentivirus, 21 μg of gag/pol, 7 μg of vesicular stomatitis virus glycoprotein, and 7 μg of shRNA plasmids were transfected into HEK293 cells using Lipofectamine 2000 (ThermoFisher Scientific). After 48 h, the packaged lentivirus particles were concentrated from the medium and the concentrated viruses were stored at −80 °C. Lentivirus particles containing shRNA were transduced into HFFs. Then, 1.5 × 10^6^ cells were plated onto 75 cm^2^ flask and 15 mL of concentrated virus and polybrene (final concentration, 8 μg/mL) were added to the cells, and incubated for 24 h. Following transduction, puromycin (2 μg/mL) was added to select for stably transduced cells. TRC control HFFs and shTNKS1+2 double knockdown HFFs were counted and an equal number of cells was plated into each well prior to infection.

### 4.11. Statistical Analysis

Statistical analyses were done using GraphPad Prism v6 (GraphPad Software, Inc. La Jolla, CA, USA) for Mac OSX. For the growth analysis, sample groups were compared to the control infected group using a one-way ANOVA. *P*-values were adjusted for multiple comparisons. For Western blot quantification, an unpaired two-tailed *t*-test with Welch’s correction was used. In all the figures, the following convention has been followed to indicate significance: * indicates *p* value < 0.05, ** indicates *p* < 0.01, and *** indicates *p* < 0.001.

## 5. Conclusions

Based on our data, we conclude that: (1) Decreased β-catenin expression in HCMV-infected cells is the final product of virus strategy to inhibit Wnt. (2) The inhibition of PARsylation activity is specific to TNKS and. (3) Chemical inactivation or knockdown of TNKS is beneficial for HCMV replication and, (4) regulation of TNKS may play an important role in HCMV replication and propagation.

## Supplementary Materials

The following are available online at http://www.mdpi.com/1999-4915/8/1/8/s1, Figure S1: XAV939 does not induce PARP1 or PARP2 transcription, Figure S2: Growth of TB40 is enhanced in the presence of XAV939, Figure S3: Sensitivity to XAV939 is cell-type specific.
