# Supplementary material for: Human Cytomegalovirus Inhibits the PARsylation Activity of Tankyrase—A Potential Strategy for Suppression of the Wnt Pathway
Source: Viruses. 2015 Dec 29;8(1):8. doi: 10.3390/v8010008 (PMC4728568; doi:10.3390/v8010008)
Supplement: Supplementary file 1 [file viruses-08-00008-s001.docx]

**Supplementary Materials: Human Cytomegalovirus Inhibits the PARsylation Activity of Tankyrase—A Potential Strategy for Suppression of the Wnt Pathway**

Sujayita Roy, Fengjie Liu and Ravit Arav-Boger *


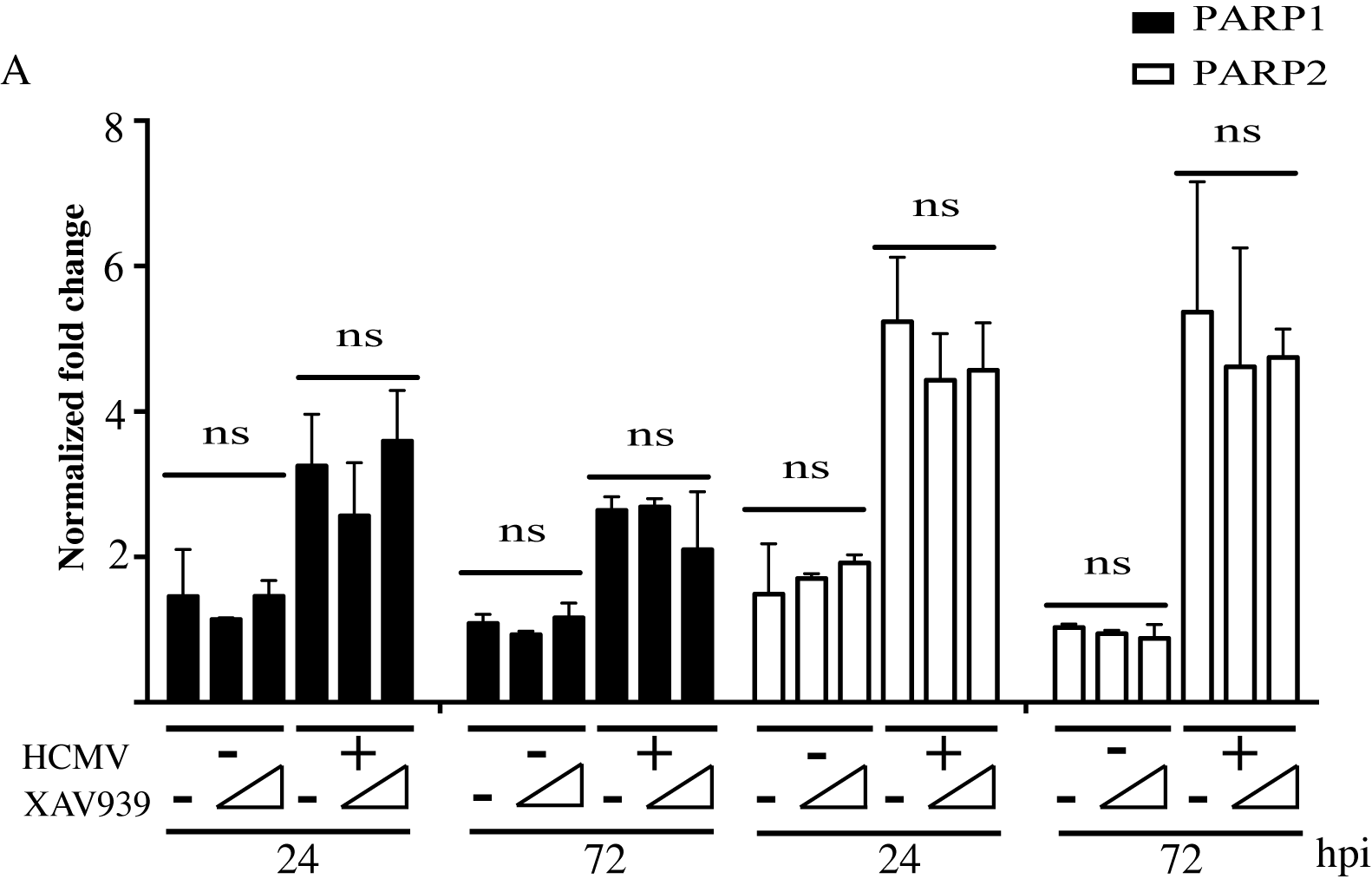


**Figure S1.** XAV939 does not induce PARP1 or PARP2 transcription. Quantitative real-time PCR showing levels of PARP1 and PARP 2 mRNA in non-infected and infected HFFs at 24 and 72 hpi, with XAV939 ( denotes increasing concentrations of XAV939, 0.1 and 1 µM). Data show average of quadruplicate wells from one experiment. ns indicates non-significance.

**
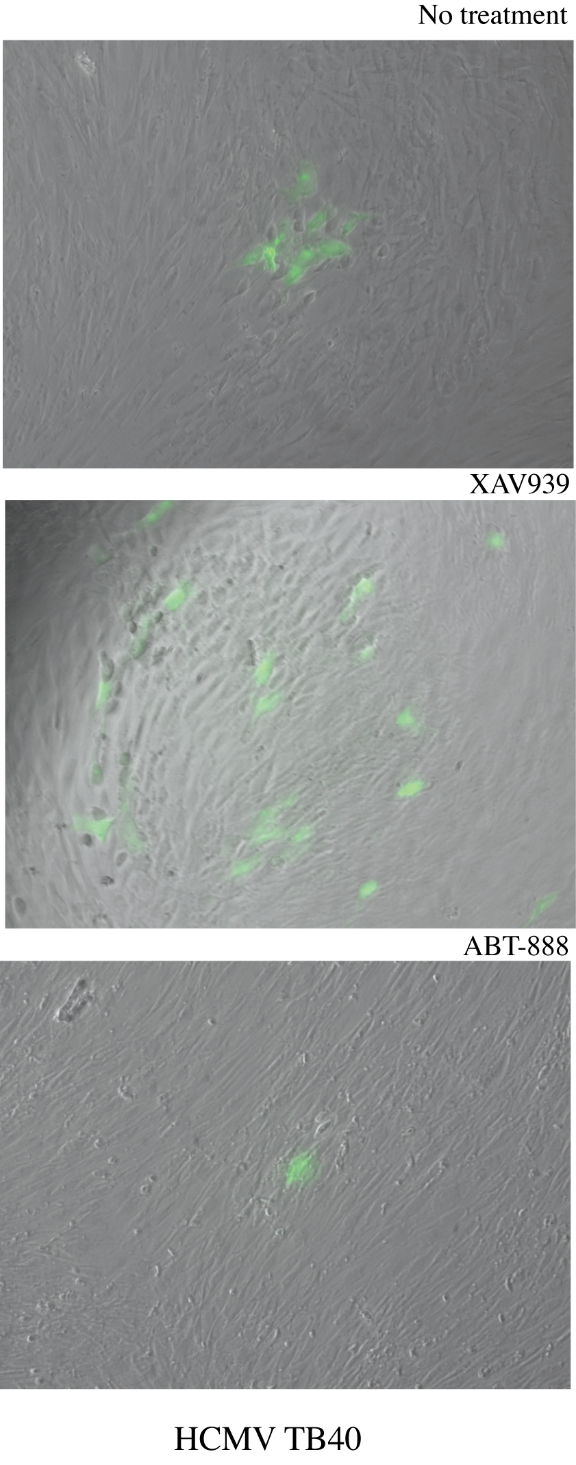
**

**
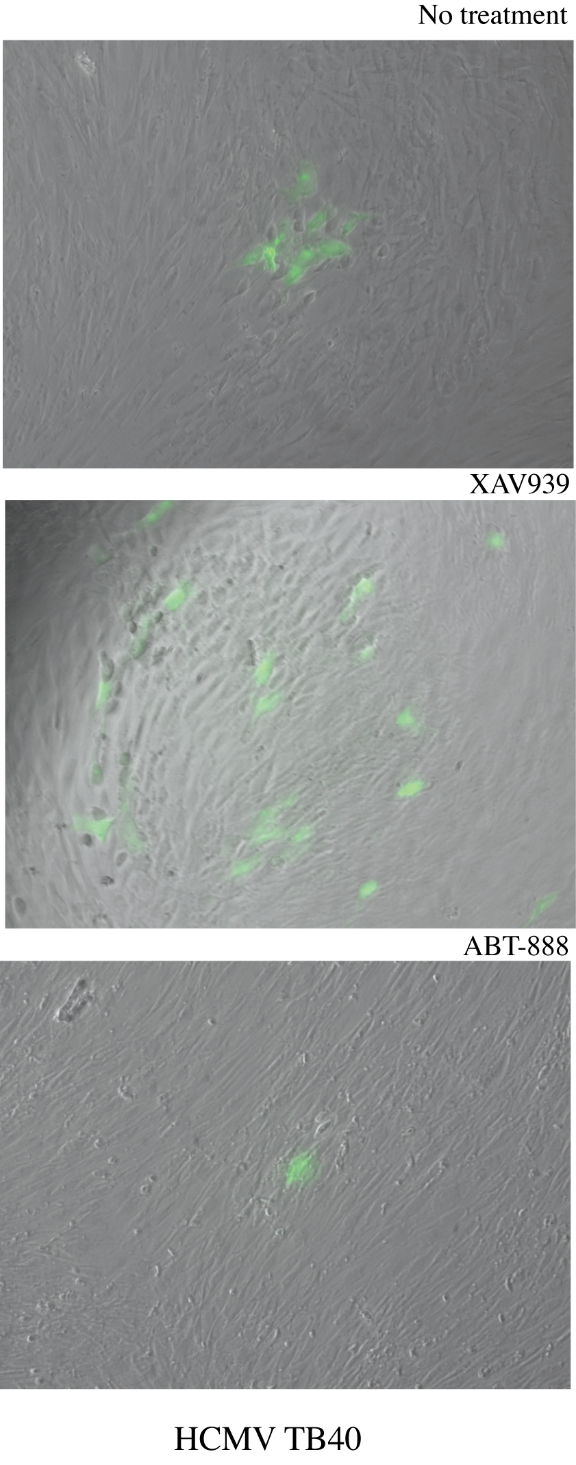
**

**Figure S2.** Growth of TB40 is enhanced in the presence of XAV939. HCMV TB40 strain was infected with XAV939 (PARP5 inhibitor), and ABT-888 (PARP1&2 inhibitor). HFFs were infected to yield approximately 50–100 plaques/well and imaged at 8 days after plating. Representative superimposed GFP fluorescence (TB40) and Differential Interference Contrast (DIC) images of HFFs are shown at 10X magnification.


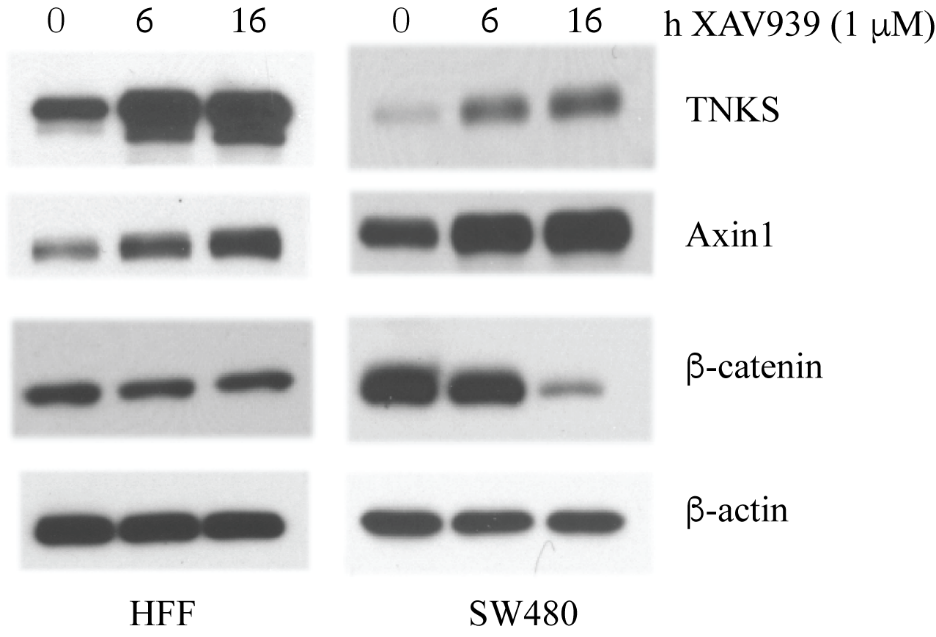


**Figure S3.** Sensitivity to XAV939 is cell-type specific. Western blot showing Wnt protein expression (TNKS, Axin1, β-catenin) in HFFs and SW480 cells after 0, 6 and 16 h of treatment with 1 µM XAV939. Data are representative of three independent experiments.

© 2015 by the authors; licensee MDPI, Basel, Switzerland. This article is an open access article distributed under the terms and conditions of the Creative Commons by Attribution (CC-BY) license (http://creativecommons.org/licenses/by/4.0/).
